# Molecular Classification and Therapeutic Targets in Ependymoma

**DOI:** 10.3390/cancers13246218

**Published:** 2021-12-10

**Authors:** Thomas Larrew, Brian Fabian Saway, Stephen R. Lowe, Adriana Olar

**Affiliations:** 1 Department of Neurosurgery, Medical University of South Carolina, Charleston, SC 29425, USA; larrew@musc.edu (T.L.); saway@musc.edu (B.F.S.); 2 Neurosurgical Associates, Knoxville, TN 37920, USA; srlowe@utmck.edu; 3 NOMIX Laboratories, Denver, CO 80218, USA

**Keywords:** ependymoma, subependymoma, RELA, YAP1, ZFTA, PFA, PFB, MYCN, Group A, Group B, myxopapillary, targeted therapy

## Abstract

**Simple Summary:**

Molecular characterization of ependymoma has revolutionized its categorization. This new molecular classification has implications particularly in targeted therapeutics. Amongst the ten subgroups of ependymoma currently described, three are found in the spinal compartment, and three in the infratentorial and supratentorial compartments respectively; the subependymoma subgroup is found in all these anatomic compartments. Each subgroup carries unique molecular features that lead to oncogenesis and to disparities in prognosis. Here, the molecular classification, key clinical features, current understanding of tumorigenesis, and potential molecular targets for cranial and spinal ependymoma are discussed.

**Abstract:**

Ependymoma is a biologically diverse tumor wherein molecular classification has superseded traditional histological grading based on its superior ability to characterize behavior, prognosis, and possible targeted therapies. The current, updated molecular classification of ependymoma consists of ten distinct subgroups spread evenly among the spinal, infratentorial, and supratentorial compartments, each with its own distinct clinical and molecular characteristics. In this review, the history, histopathology, standard of care, prognosis, oncogenic drivers, and hypothesized molecular targets for all subgroups of ependymoma are explored. This review emphasizes that despite the varied behavior of the ependymoma subgroups, it remains clear that research must be performed to further elucidate molecular targets for these tumors. Although not all ependymoma subgroups are oncologically aggressive, development of targeted therapies is essential, particularly for cases where surgical resection is not an option without causing significant morbidity. The development of molecular therapies must rely on building upon our current understanding of ependymoma oncogenesis, as well as cultivating transfer of knowledge based on malignancies with similar genomic alterations.

## 1. Introduction

Ependymomas are primary central nervous system (CNS) tumors derived from the ependymal lining of the ventricular system [1,2]. These tumors are found in both the pediatric and adult population and are found throughout the CNS, including the supratentorial (ST) space, the infratentorial (IT) space and the spinal (SP) compartment. They are most commonly found in the ventricular system, but also in the parenchyma.

Based on the Central Brain Tumor Registry of the United States, ependymal tumors represent approximately 1.6% of all primary CNS tumors. Incidence of ependymal tumors ranges from 0.29 to 0.6 per 100,000 person-years and is lowest in the first two decades of life and highest in the 65–74 years old age group [3]. Tumor location is largely dependent on the patient age, with nearly 90% of pediatric ependymal tumors occurring intracranially, and approximately 65% of adult tumors occurring in the spine [4]. Amongst patients with intracranial ependymomas, there is significant morbidity and mortality. In a multicenter study of 282 adult patients, the 5-year overall survival (OS) rate was 62% for ST tumors and 85% for IT tumors [5]. Patients with SP ependymomas fare better with a 5-year OS of 97%.

Management of adult patients with ependymoma involves surgical resection for its cytoreductive effects and, in many cases, to restore normal cerebrospinal fluid (CSF) dynamics [6,7,8]. Postoperative radiotherapy is usually employed, particularly in cases of World Health Organization (WHO) grade II intracranial ependymoma after subtotal resection (STR) and WHO grade III intracranial and spinal anaplastic ependymoma regardless of the extent of resection [5,8,9,10,11]. The use of radiotherapy remains controversial for WHO grade II intracranial ependymoma and spinal ependymoma after gross total resection (GTR) [12]. Chemotherapy is typically reserved for cases of advanced or recurrent ependymoma that cannot be further resected or irradiated [11]. There is very little description of efficacious use of chemotherapies for ependymoma in general. In a study of adult recurrent low-grade and anaplastic SP, ST, and IT ependymoma, a regimen of temozolomide and lapatinib was used with modest results and a median progression-free survival (PFS) of 8 months [13]. Among the small number of studies looking at the use of chemotherapy for ependymoma, there is a lack of any standard regimens with durable results, though use of platinum compounds or temozolomide has been suggested due their favorable safety profiles [11,13,14,15,16,17,18]. There has been limited use of immunotherapy for adult ependymoma, though there are a few documented cases of its use in pediatric recurrent ependymoma [19].

The management of pediatric patients with ependymoma follows a similar paradigm except for several key differences. Pediatric ependymoma management centers around GTR and radiotherapy. Based on National Cancer Institute recommendations, all intracranial pediatric ependymoma cases require surgery and postoperative radiotherapy unless the patient is under 1 year of age [20]. The European Association of Neuro-Oncology (EANO) has similar recommendations except it is recommended that postoperative radiotherapy should be used in children older than 18 months, or in children between 12 and 18 months with significant neurological deficits [11]. Traditionally, chemotherapy regimens were utilized in children younger than 3 years of age with newly diagnosed ependymoma to defer radiation. However, there is mounting evidence that earlier radiation may lead to improved outcomes [21,22,23] In comparing the trials POG-9233 trial and ACNS0121, which had similar protocols except for the use of radiotherapy, there was a 50–60% improvement in survival for patients under 3 years old who were treated with radiation therapy [24,25]. It is recommended that second-look surgery be performed when GTR is achievable, as it leads to better disease control [26]. In STR cases, the benefit of pre-radiation chemotherapy is still being investigated but may be used [27]. Pediatric patients with disease recurrence should undergo resection with postoperative radiotherapy [28]. Chemotherapy has been used in recurrent cases but the response generally lacks durability [29,30]. Chemotherapy regimens should be based on prior exposures, though clinical trials should be explored [11].

The management strategy of adult patients that suffer from relapse is currently evolving as our understanding of the molecular drivers of this disease are being further investigated [31]. Moreover, the most effective management of disseminated versus local recurrence is also not well delineated. Although there is a dearth of randomized clinical data, there is a consensus that recurrent lesions that are non-operable should receive radiotherapy in both the adult and pediatric populations. The utility of chemotherapy and immunotherapy for disease recurrence is also actively being explored. EANO recommends platinum or temozolomide based on their favorable toxicity profiles, though clinical trials should be considered [11,17]. Ongoing research on this topic includes locoregional delivery of chimeric antigen receptor T (CAR T) cells targeting various surface antigens expressed on recurrent ependymomas, which has been shown to be highly efficacious in a mouse model [32]. As our treatment modalities improve for this pathology, and longevity of this patient population subsequentially increases, optimization of treatment regimens for recurrence using a combination of surgery, radiation, and chemo-/immuno-therapies will need to be further investigated.

## 2. History

Ependymomas have classically been diagnosed based on the WHO histological classification strategy, which was designed to integrate histopathological characteristics to facilitate tumor diagnosis, guide treatment strategy, allow for prognostication, and aid research by allowing for interlaboratory comparisons. In 2016, the WHO classification for CNS tumors incorporated not only light microscopy, immunohistochemical lineage-associated proteins, and ultrastructural characteristics, but also a few molecular markers, namely *RELA*-fusion ependymoma, to enhance the diagnostic and prognostic utility of this classification strategy [1]. Despite this, controversy in the field remains regarding the use of primarily histology to guide tumor grading. This controversy stems from the notable histopathologic variations seen between and within specimens, the large inter-observer grading discrepancies among neuropathologists, and the lack of consistent prognostication based on WHO grading [33,34,35,36]. There was a clear paucity amongst scientists and clinicians on how to effectively categorize this diverse CNS pathology to better identify, analyze, and prognosticate in order to provide patients with accurate information regarding their diagnosis. Similar to other CNS malignancies, investigators began to focus on molecular signatures as a means to fill this void.

The introduction of molecular markers in the 2016 WHO classification and the expansion in the 2021 WHO classification, was a function of several investigations elucidating the genetic uniqueness within the histologically defined subgroups of ependymomas. These findings began as a series of multi-omics studies that separated genomic groups of ependymomas with clinical and prognostic correlation. Early studies focused on gene expression and copy number profiles, while late studies focused on epigenomics [37,38,39,40,41,42,43,44].

In 2015, a landmark clinical and genomic study by Pajtler et al., utilized DNA methylation profiling of 500 ependymomas to identify nine subgroups [40]. This project solidified and supported previous research emphasizing that despite similar histopathological features among ependymoma tumors, their diverse biological behavior could be better defined and categorized based on genomic markers. The nine subgroups of ependymomas were divided evenly amongst the three major compartments of the CNS. The SP compartment contained the subgroups of SP subependymoma (SP-SE), SP myxopapillary ependymoma (MPE), and SP ependymoma (SPE), all of which have predilection for the adult population. The IT compartment contained the subgroups posterior fossa subependymoma (PF-SE), posterior fossa ependymoma Group A (PFA), and posterior fossa ependymoma Group B (PFB), of which, the PFA alone has a strong preponderance for the pediatric population. Lastly, the ST compartment contained the subgroups of ST subependymoma (ST-SE), ST ependymoma with *YAP1* fusion (ST-YAP1), and ST ependymoma with *RELA* fusion (ST-RELA). All nine subgroups were found to have distinct DNA methylation patterns that correlated with pertinent clinicopathological variables, such as age and sex. The study was also able to demonstrate that risk stratification by molecular subgrouping is superior to the histopathological grading used by the 2007 WHO grading system. However, it is important to note that in this and following studies, many classic histology ependymomas (grade II) were classified into the SE and MPE subgroups [37,40]. Nevertheless, this study served as the foundation for subsequent studies validating and expanding upon these new molecular subgroups of ependymomas.

In 2021, the WHO Classification of Tumors of the CNS will be again updated with its 5th edition attempting to capture all of the recent updates in genomic profiling and to address some of the aforementioned issues. This update introduced the *C11orf95*/*ZFTA*-fusion subgroup (same as the former 2016 *RELA*-fusion subgroup), *YAP1*-fusion subgroup, PFA subgroup, PFB subgroup, and the SP ependymoma with *MYCN* amplification subgroup. These were added as unique genomic markers, and transcriptional patterns were identified that could better differentiate ependymomas despite similar histological features [45,46,47,48,49,50]. While this offers additional molecular categories, data is lacking to assign grading based solely on molecular alterations, as is seen in diffuse gliomas [49,50].

**Figure 1:**
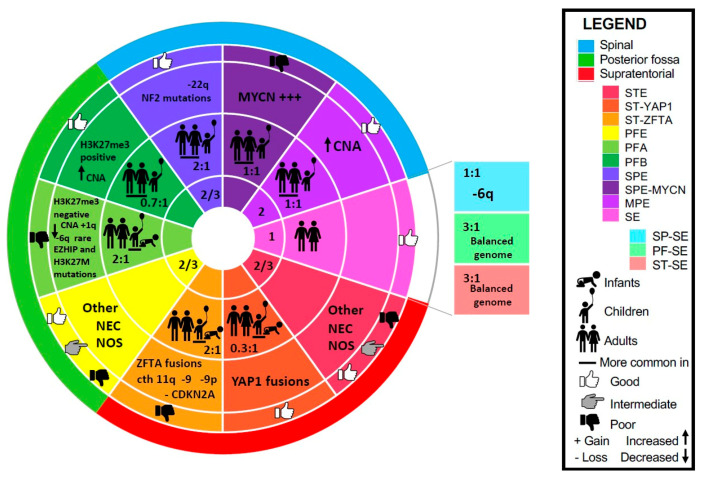
Ependymoma molecular classification per WHO 2021. Concentric discs represent (inner to outward): WHO grade, age and sex ratio (male to female), characteristic molecular features, outcome, location. Abbreviations: CNA—copy number alterations, cth—chromothripsis, MPE—myxopapillary ependymoma, NEC—not elsewhere classified (other molecular alteration, not described), NOS—not otherwise specified (molecular testing is not available), PFE—posterior fossa ependymoma, PFA—posterior fossa ependymoma Group A, PFB—posterior fossa ependymoma Group B, PF-SE—posterior fossa subependymoma, SE—subependymoma, SP-SE—spinal cord subependymoma, SPE—spinal cord ependymoma, SPE-MYCN—spinal cord ependymoma with *MYCN* amplification, STE—supratentorial ependymoma, ST-SE—supratentorial subependymoma, ST-YAP1—supratentorial ependymoma with *YAP1* fusion, ST-ZFTA—supratentorial ependymoma with *ZFTA* fusion.

The 2021 WHO classification, the Pajtler study, and the subsequent studies that examined these distinct subgroups in the population, their underlying oncogenic drivers, as well as proposed molecular targets, are the subject of this review. Besides, SE and MPE which remain their own histological categories, ependymomas are now primarily defined by anatomic location (SP, PF, ST), histology (grade 2 or 3), and molecular features [49,50]. In this review, we describe the three subgroups of ependymoma found in each of the three anatomic compartments, as well as the SE subgroups found at every location. These 10 subgroups and their characteristics are summarized in Figure 1.

## 3. Subependymoma (SE)

**Figure 2:**
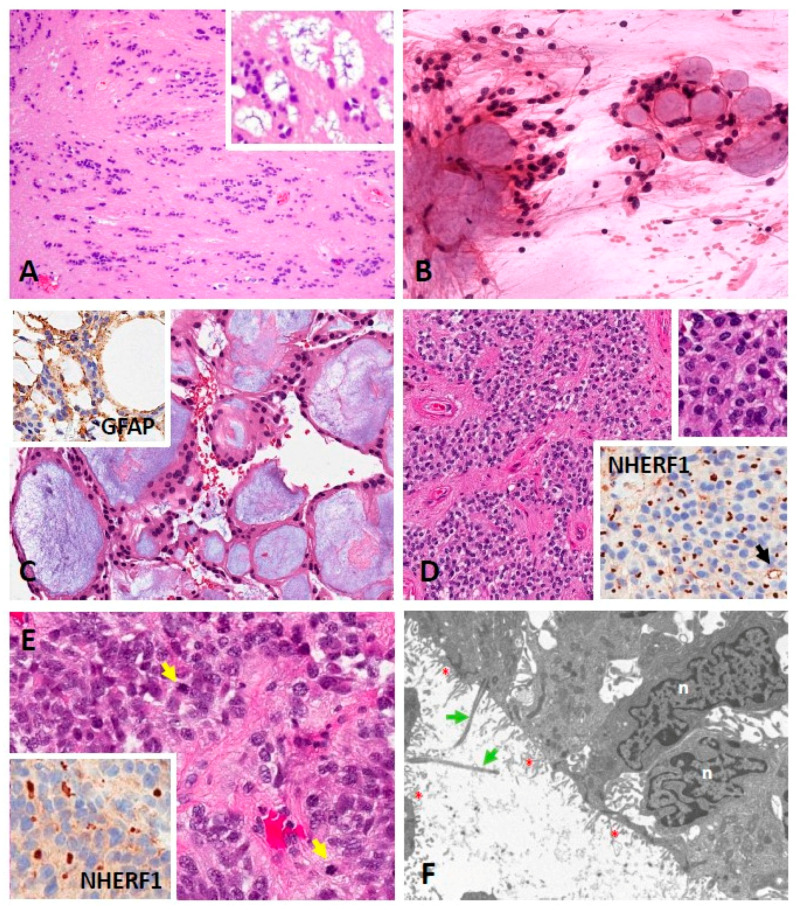
Morphology of subependymoma and ependymoma. (**A**) Subependymoma composed of clusters of isomorphic nuclei embedded in a fibrillary matrix of glial cell processes (HE, 100×). Cystic degeneration and nuclear pleomorphism (degeneration-related) can be present in larger tumors (inset) (HE, 200×). (**B)** Myxopapillary ependymoma showing ependymal cells with bland oval nuclei and fibrillary processes around aggregates of myxoid material (smear, HE, 200×). (**C**) Tissue section of myxopapillary ependymoma showing bland GFAP positive (inset, GFAP, 200×) ependymal cells around blood vessels with myxoid degeneration (HE, 200×). (**D**) Ependymoma composed of neoplastic glial cells arranged around blood vessels in pseudorosettes (HE, 100×). The tumor nuclei are round to oval, monomorphic with stippled chromatin (top inset) (HE, 200×). Anti-NHERF1/EBP50 immunohistochemistry showing numerous dot-like microlumens and a rare ring-like structure (arrow) (bottom inset) (200×). (**E**) Anaplastic ependymoma with enlarged pleomorphic nuclei and mitotic activity (arrows) (HE, 200×). Anti-NHERF1/EBP50 immunohistochemistry showing an area with microlumens (inset) (200×). (**F**) Electron micrograph showing the lumen of a true rosette lined by ependymal tumor cells with rare cilia (green arrows) and abundant microvilli (red asterisks) (18,000×). Abbreviations: HE—hematoxylin and eosin, n—nucleus Note: Photographs in D-F were kindly provided by Dr. Maria-Magdalena Georgescu, NeuroMarkers PLLC, Houston, TX, USA.

In the 2021 CNS WHO classification, subependymoma (SE) remains a separate entity (encompassing SP-SE, ST-SE and PF-SE–described below) identified by morphologic criteria (Figure 2A) as there is currently no clinical justification of SE molecular subgroups at each anatomic site [49,50]. Grossly, these tumors are tan-white-gray, nodular, and firm, bulging into the ventricles. The histopathology is defined as clusters of isomorphic nuclei embedded in a dense, fine, glial fibrillary background, mild nuclear pleomorphism, microcystic formations (especially in lateral ventricular tumors), with or without occasional hemorrhage, and/or calcification. SE express glial acidic fibrillary protein (GFAP) and ezrin-radixin-moesin binding protein 50 or Na+/H+ Exchanger Regulatory Factor (NHERF1/EBP50) microlumens, whereas epithelial membrane antigen (EMA) is rarely expressed [1,51,52]. They are considered slow growing WHO grade 1 tumors often presenting with symptoms related to CSF obstruction if intracranially located, given their predilection for the ventricular system. They can present with spinal cord/nerve root compression if located in the spine.

### 3.1. Spinal Subependymoma (SP-SE)

Spinal subependymomas (SP-SE) have a predilection for the cervical or cervicothoracic junction, and typically present with insidious myelopathy or radicular pain [53]. This subgroup commonly presents in the adult population with a mean age of presentation of 44 years old, and with an even sex distribution, based on the few case series that have been published [54,55].

Patients with SP-SE have an excellent prognosis when treated with the standard of care treatment strategy of microsurgical resection [55]. GTR with minimal disruption of adjacent vital structures via microsurgery is the gold standard treatment, and is often feasible with the advancement of microsurgical tools and intraoperative neuromonitoring. When GTR is not achievable given proximity to eloquent structures, STR is advised with serial follow-up imaging. A large systematic review assessing outcomes in 105 cases of SP-SE found that 65% of patients underwent GTR and that of these, 57% had worsened function after surgery, while only 41% of the patients that underwent STR or partial resection experienced worsened function [55]. No patients had tumors with malignant transformation; therefore, long-term survival for all patients was expected. This study emphasizes the significant morbidity associated with surgical intervention of this subtype of tumor given the eloquent adjacent structures despite the excellent mortality rates. The authors make the argument for the benefits of cure in this disease. However, when the demarcation margin is not very clear during surgical debulking, the benefits of cure via GTR may not outweigh the risks of causing significant neurological morbidity as disease progression is likely to be very slow. No adjuvant radiotherapy is needed as good clinical outcomes are seen following both gross and STR of SP-SE [56]. Altogether, while SP-SE have an excellent prognosis when treated with microsurgical resection, there is inherent risk taken when surgically removing these tumors from the eloquent spinal cord, which may limit therapeutic reach.

Research into the molecular drivers underlying oncogenesis of SP-SE has been sparse, and likely secondary to its benign clinical course. The two largest genetic analyses of this tumor subgroup both showed loss of 6q [37,40]. In addition, Witt et al. noted an almost 50% copy number reduction in 19p and 19q, while in the seven SP-SE patients in the Pajtler study no chromosome 19 copy number loss was noted. The one, uniformly defining characteristic of this subgroup is deletion of chromosome 6q [40]. Previously, the role of 6q deletion in all subsets of ependymoma has been controversial as studies have reported variable influence on patient prognosis. Specifically, 6q25.3 deletions were found to be markers of good prognosis and survival, as shown by the risk stratification scheme of intracranial ependymomas proposed by Korshunov et al. and confirmed in the pediatric population by Monoranu et al. [57,58]. Conversely, Rajaram et al. found 6q23 deletions to be a marker of disease progression in a mixed population of ependymoma subgroups [59]. While the role of 6q deletion in SE tumorigenesis and behavior requires further research, its involvement and targeting in other malignancies may provide avenues for future treatment strategies. Specifically, the c-myeloblastosis gene (*c-MYB*), a proto-oncogene encoding a transcription regulator that plays an essential role in regulation of hematopoiesis and located on chromosome 6q23.3, is being explored as a driver in the oncogenesis of this tumor [60]. Additionally, 6q cytogenetic abnormalities are commonly seen in T-cell lymphoblastic leukemia/lymphoma. Research in this field has recently identified *EPHA7*, a tumor suppressor gene found on 6q that encodes the protein Ephrin type-A receptor 7, a receptor tyrosine kinase that mediates developmental events in the nervous system [61]. *EPHA7* may contribute in part to the onset of T-cell lymphomas.

### 3.2. Posterior Fossa Subependymoma (PF-SE)

The histopathologic origin of SE in the IT compartment remains uncertain. Possible cell origins include ependymal-glial precursor cells, subependymal plate cells, and tanycytes [62,63]. Macroscopically, the tumors are firm, well-delineated, and lobulated masses bulging into the fourth ventricle (IVthV). The most common location for intracranial SE. PF-SE is typically found on the floor or the roof of the IVthV. Approximately 20% of these tumors are calcified. Unlike ST-SE, PF-SE is unlikely to be cystic. Dissimilar to most other ependymoma subgroups, PF-SE occurs in middle-aged to older patients, with the average age around 60 years old [40,62]. PF-SE is more common in the male population with almost a 3:1 ratio [62].

PF-SE carries low pathogenicity and, in fact, is often not discovered until postmortem examination [64,65]. GTR is generally thought to be curative, though recurrences can occur [62,66]. Radiotherapy may be used in cases of STR, but is typically reserved as salvage therapy if progression occurs. Chemotherapy and molecularly targeted therapy have not routinely been employed given the tumor’s slow growth and low risk for recurrence or metastasis. The 5-year PFS and OS are generally high, at 86–90% and 91–100%, respectively [40,62,67].

Genomic analyses of PF-SE have had mixed results regarding copy number variations [37,40]. While the landmark paper by Pajtler et al. did not find any copy number loss or gain, there was over 50% copy number loss in chromosome 19p and 19q in the 24 PF-SE patients studied by Witt et al. Based on examination of the transcription profiles of PF-SE tumors, its tumorigenesis appears to be related to fatty acid metabolism, mast cell and leukocyte processes (KIT), signal transduction pathways (specifically STAT), as well as, chemotaxis [40]. In a study by Kong et al., an SE cell line was created and a tissue microarray analysis was performed demonstrating high tumor expression levels of topoisomerase II-β, HIF-1α, E3 ubiquitin-protein ligase Mdm2, and nucleolin on immunohistochemistry and Western blot analyses [68]. Topoisomerase II-β is encoded by *TOP2B* and is a key decatenating enzyme [69]. HIF-1α is encoded by *HIF1A* and functions as a master transcriptional regulator in the response to hypoxia. E3 ubiquitin-protein ligase Mdm2 is encoded by *MDM2* and functions to mediate ubiquitination of p53 leading to its degradation. Nucleolin is encoded by *NCL* and is a major nucleolar protein associated with intranucleolar chromatin and preribosomal particles that induces chromatin decondensation by binding to histone H1. Given the benign course of PF-SE, there are no chemotherapy regimens commonly used, nor are there any current targeted therapies for this disease. At the time of writing, there are no clinical trials for chemotherapies or molecularly targeted therapies for SE. *KIT* may be a viable target as it is only expressed at high levels in PF-SE tumors but not the other subgroups [40]. Multikinase inhibitors, such as imatinib, have been demonstrated to inhibit the autophosphorylation of c-KIT and could play a role in disease progression [70,71]. Topoisomerase inhibitors and p-STAT3/HIF-1α inhibitors appear to inhibit SE cell line growth suggesting a topoisomerase II inhibitor, such as FDA-approved etoposide and teniposide, could counteract PF-SE disease progression [68].

### 3.3. Supratentorial Subependymoma (ST-SE)

Supratentorial subependymoma (ST-SE) is often attached to the wall of the ventricular system. While the IVthV is a common intracranial location (40–60% of intracranial cases), SE is most commonly found supratentorially in the lateral ventricles (30–45% of intracranial cases), as subependymal glial cells are thought to play a large role in the origin of this neoplasm [72,73,74]. Similar to its IT counterpart, patients can present with symptoms related to CSF obstruction [67,75]. Presentation is common throughout adulthood but is most frequently seen in the third to sixth decades of life and in males rather than females [40,65,67,76,77].

The current gold standard treatment is maximal resection [67,76]. The use of adjuvant radiotherapy is generally not recommended as a good prognosis with surgery alone is seen if GTR is achieved [40,62,66,67]. Similar to its IT counterpart, there may be a role for radiotherapy if there is recurrence or symptomatic residual disease [67,76]. Unlike PF-SE, GTR may be more easily achievable given ST-SE is not in proximity to the brainstem. OS is excellent, at 96–100% for 1, 5, and 10-year [40,78].

Because of the uncommon nature of this subgroup, as well as the excellent prognosis following GTR, there has been little research regarding molecular targets for ST-SE. Similar to SP-SE and PF-SE, the two largest genomic studies have shown disputing results regarding copy number variations [37,40]. The Pajtler analysis of 21 ST-SE patients demonstrated a balanced genome, while the Witt analysis of 14 ST-SE patients demonstrated a 50% copy number loss in chromosome 19p and 19q. This discrepancy will have to be solved by future studies in all SEs. Regarding target development, the previously mentioned study by Kong et al. sought to identify and prioritize potential therapeutic targets for SE tumors through the use of tissue microarrays, ex vivo analysis, and in vitro cytotoxic assays [68]. This study derived the first-known human SE cell line from a resected SE and has laid the foundation for future research to identify potential molecular targets and develop therapeutic approaches. Of importance, this study demonstrated tumor expression of p53, MDM2, HIF-1α, topoisomerase II-b, p-STAT3 and nucleolin while also showing growth suppression of SE cells ex vivo utilizing a topoisomerase inhibitor (WP744) and the p-STAT3/HIF-1α inhibitors (WP1066 and WP1193). The targets highlighted in this molecular study may be applicable to SP-SE, IT-SE, and ST-SE; however, given the benign course of SE, development of targeted therapy may have a limited role in management in comparison to other ependymoma subgroups that may have malignant transformation. In rare instances of symptomatic residual or inability to resection, particularly in SP-SE cases, there may be a role for targeted therapies, but given the rarity of this, the authors encourage investigation into other ependymoma subgroups.

## 4. Myxopapillary Ependymoma (MPE)

Myxopapillary ependymoma (MPE) is a tumor originating almost exclusively in the spine (conus medullaris, cauda equina, and filum terminale). MPE was previously classified as WHO grade I in the 2016 WHO CNS classification, but because there is evidence that its outcomes are comparable to those of classic ependymoma, the new 2021 WHO CNS classification recommends assigning grade 2 to MPE [5,49,50]. MPE is defined by slow growth and favorable prognosis and accounts for 27% of all SP ependymoma [79]. Grossly, MPE are often encapsulated, lobulated, tan, and soft. They have a glistening cut surface with or without cyst formation and/or hemorrhage. Histopathologically, MPE is composed of well-differentiated cuboidal to elongated tumor cells radially oriented around hyalinized fibrovascular cores, commonly with degeneration-derived myxoid accumulation (Figure 2B,C) [1,80,81,82]. Patients frequently present with radicular pain and/or back pain [83]. While these tumors are found within all age groups, they are commonly found in the third and fourth decade of life, with a slight male predominance [84].

MPE is retained as a separate entity in WHO 2021 and defined by histological criteria. Methylation analysis by Pajtler et al., separated a MPE methylation subgroup from two SE and six classic ependymoma methylation subgroups [40]; however, that study, and an additional methylation analysis in a different study, grouped many classic histological ependymomas in the MPE and SE methylation subgroups, suggesting that methylation classification might not be reliable [37,40]. Moreover, such classification does not bring additional clinical significance to MPEs [49,50].

Similar to SP-SE, the gold standard treatment modality for MPE is maximal safe resection [85]. A systematic review of 28 articles demonstrated that overall recurrence rate after GTR (16%) was significantly lower than what was demonstrated following STR (33%) in the pooled cohort, with a mean follow-up of 75 months [84]. This review also demonstrated that adjuvant radiotherapy is not necessary as is not associated with a decrease in recurrence. There are reports, however, that radiotherapy may aid in disease control. An MD Anderson Cancer Center study showed that the addition of adjuvant radiotherapy to surgery was associated with significantly higher 10-year PFS rates (75% for surgery and postoperative radiotherapy vs. 37% for surgery alone) and higher 10-year local tumor control rates (86% for surgery and postoperative radiotherapy vs. 46% for surgery alone) [86]. A 10-year OS of 75–100% has been demonstrated with STR followed by radiotherapy [87,88].

The current state of understanding of the oncogenic drivers that lead to MPE is still in its infancy as the excellent prognosis with surgery has not necessitated targeted therapies. While chromosomal instability is considered to be the defining genomic characteristic of this subgroup of ependymoma, as seen by the copy-number variation gains observed by Pajtler et al. in their DNA methylation array of 26 samples of MPE, there are three notable genes that are areas of current research and possible future molecular targets [40]. Overexpression of *homeobox B13* (*HOXB13*), a gene encoding a transcription factor that regulates skin development, has been shown by multiple studies [40,89]. The key involvement of this gene in developmental pathways has led researchers to hypothesize it plays a role in MPE tumorigenesis. Confounding this hypothesis is that the upregulated HOX genes commonly seen in MPE are *HOXB13*, *homeobox A13* (*HOXA13*), *homeobox C10* (*HOXC10*), and *homeobox D10* (*HOXD10*), all of which are genes overexpressed in the developing lumbar spine [90]. Immunohistochemistry analysis of adult filum terminale did not demonstrate HOXB13 expression, supporting the hypothesis that HOX groups 10–13 are expressed in early development and switched off once segmentation has completed, and its presence in adult MPE represents aberrant expression [89]. Oncogenesis amongst HOX genes is a growing area of research as aberrant HOX expression has been demonstrated in other malignancies including acute myeloid leukemia, breast, cervical, small-cell and non-small cell lung, prostate, skin, and thyroid cancers [89]. HOXB13 protein overexpression must be recognized and further assessed as a potential future therapeutic target [89,91]. Two other genes with overexpression seen in MPE are *neurofilament light chain* (*NEFL*), encoding a Class IV intermediate neurofilament expressed in neurons and located on chromosome 8p21.2 in close proximity to transcription factor binding sites of HOX genes, and *platelet derived growth factor receptor alpha* (*PDGFRA*), a gene which encodes a tyrosine kinase [89,92]. While the ubiquitous expression of these genes in various processes throughout the CNS has thus far precluded any significant progress in developing targeted therapies, as seen by the lack of clinical trials addressing these overexpressed genes, these are promising targets for future research. Lastly, a recent study assessing DNA methylation and gene expression profiles of pediatric SP ependymomas also identified overexpression of *HOXB13*, lending further evidence towards the importance of this gene [93]. This study also demonstrated significant overexpression of genes involved in the mitochondrial oxidative phosphorylation respiratory chain, such as *cyclooxygenase-2* (*COX2*), a gene that encodes for cyclooxygenase, which plays a vital role in inflammation and has been shown to be overexpressed in various cancers as it also plays a role in cell proliferation, neovascularization, and tumor metastasis [93,94,95,96]. While this was a small series in the pediatric population, it has provided more insight into the complex molecular amalgam that drives the tumorigenesis of MPE. As surgical resection remains the mainstay treatment of those afflicted by this tumor, further research will attempt to identify key genes that are uniquely expressed that may be future targets.

## 5. Spinal Ependymoma (SPE)

Spinal ependymoma (SPE) (WHO grade 2 or 3) is a tan, soft, well-circumscribed tumor composed of monomorphic glial cells with round to oval nuclei with speckled chromatin. They form perivascular pseudorosettes and/or true ependymal rosettes. When hypercellularity, cellular atypia, frequent mitoses, abundant endothelial proliferation and/or palisading necrosis are present, the tumor is deemed to be WHO grade 3. Histological patterns (clear cell, papillary, tanycytic) can be seen but do not have clinical significance [1,49,50,97]. Ependymomas express GFAP, S100 protein, EMA, and NHERF1/EBP50. The latter is a protein involved in epithelial morphogenesis and is superior to EMA for diagnosis of complex cases or ambiguous tumors (AO unpublished observations) [51]. Ultrastructurally, ependymomas are characterized by cilia, blepharoblasts, microvilli, and junctional complexes (Figure 2D–F) [1]. SPE is associated with *neurofibromatosis type 2* (*NF2*) mutations or deletions as seen in the molecular study by Pajtler et al., which found 90.5% of samples categorized into the SP ependymoma group demonstrated copy number variations of chromosome 22q where this gene is located [40].

Unlike MPE and SE, which are easily distinguishable entities, the 2016 WHO classification of ependymoma into grades II or III is less consistent [1,98]. Studies have found there to be significant intratumoral heterogeneity and, moreover, significant grade interrater variability among neuropathologists [33,99]. Although there are a number of studies demonstrating prognosis correlating with WHO grade, there is controversy regarding the prognostic utility of WHO grading of ependymoma, as there are also studies demonstrating no significant difference in survival between cases stratified per WHO grade [99,100,101,102,103]. It is for this, and the aforementioned reasons, that the field has moved towards molecular characterization of ependymoma. This tumor can be found anywhere in the spinal column but is often found in the cervical spine, which leads to the common presenting symptoms of radicular pain, myelopathy, and neck pain [97]. Men are found to be more affected than women. This subgroup is frequently diagnosed in patients 30–40 years old [37,40].

The standard treatment modality is microsurgical resection with the goal of GTR with minimal normal tissue disruption; however, the role of adjuvant radiotherapy is a topic of debate. One series of 104 (101 grade II and 3 grade III) SPEs that underwent surgical resection found the median PFS of those that underwent microsurgical resection to be 14.9 years for grade II and 3.7 years for grade III SPEs. Furthermore, they reported no significant change in PFS for patients that underwent adjuvant radiotherapy following STR [104]. Another large case series assessing long-term outcomes of 88 patients with SPE that underwent microsurgical resection, with and without adjuvant radiotherapy, found the surgical extent of resection to be an independent predictor of longer PFS, while postoperative radiotherapy after incomplete resection did not significantly correlate with longer times to recurrence [105]. A more recent study of 69 patients with SPE found for grade II lesions, STR and radiotherapy yielded better outcomes than STR alone, with a 10-year PFS of 77% and 68%, respectively [106]. Altogether, there is moderate evidence that adjuvant radiotherapy should be considered for patients that undergo STR.

Despite this excellent prognostic profile, the presence of *NF2* alterations in other CNS tumors has led to extensive research being performed to better understand the function and oncogenesis that occurs in tumors harboring this genomic variation. *NF2* is a tumor suppressor gene, located on chromosome 22q12.2 and codes for the protein Merlin. Deletions or loss-of-function mutations of this gene leads to neurofibromatosis type II, which is inherited in an autosomal dominant pattern [107]. Merlin is a scaffolding protein that links F-actin, transmembrane receptors, and intracellular effectors that modulate receptor mediated signaling pathways controlling cell proliferation and survival. The vast number of signaling pathways that are affected by Merlin emphasize its importance in integrating these pathways to influence cell morphology, motility, proliferation and survival. While Merlin’s role in all of these pathways is still not completely understood, there are three molecular pathways that have been well defined and provide insight into how loss-of-function of Merlin can lead to oncogenesis. First, Merlin serves to inhibit various membrane receptors and the RhoGTPase family signaling cascade. Through binding to the CD44 cell membrane protein, Merlin negatively regulates this protein, which functions to increase cell proliferation. Additionally, through interaction with P21 Activated Kinase (PAK), Merlin inhibits Rac Family Small GTPase 1 (Rac1)/Cell Division Cycle 42 (Cdc42) signaling which leads to downstream inhibition of effectors including Rat sarcoma virus (RAS), Phosphoinositide 3-kinase (PI3K), and Ras-related C3 botulinum toxin (RAC). This ultimately leads to reduction in downstream Rapidly Accelerated Fibrosarcoma (RAF)/Myocyte Enhancer Factor (MEF)/Extracellular Signal-Regulated Kinase (ERK), Mammalian Target of Rapamycin Complex 1 (mTORC1), and Focal Adhesion Kinase (FAK) signaling [108]. Second, through binding PI 3-Kinase Enhance-L (PIKE-L), Merlin regulates and inhibits the PI3K/Protein Kinase B (AKT) pathway, which influences and promotes cellular survival and growth. Lastly, through the mammalian Hippo pathway, Merlin is involved with inhibition of Yes-associated Protein (YAP), a protein responsible for cell proliferation control and which has important regulatory functions in regeneration, organ development and stem cell self-renewal [109]. In this pathway, Merlin functions to promote translocation of Large Tumor Suppressor Kinase 1 and 2 (LATS1/2) to the nucleus while also inhibiting Cullin Ring Ubiquitin Ligase 4 (CRL4), both leading to reduced transcriptional output of YAP and other domain transcription factors [109].

As robust research has allowed for the meticulous understanding of the role of Merlin in oncogenesis, there have been several treatments under investigation targeting these molecular pathways. Small molecular MEK inhibitors are currently under investigation as they seek to target and inhibit the RAF/MEK/ERK pathway for *NF2*-associated tumors (NCT02639546, NCT03095248 on www.clinicaltrials.gov, (accessed on 9 November 2021)). A phase 2 clinical trial is also looking at *NF2* specific molecular targets via a FAK inhibitor on *NF2* mutant meningiomas (NCT02523014/A071401). Another *NF2* specific molecular pathway target is verteporfin a benzoporphyrin derivative that is currently used as a photosensitizer in macular degeneration, as it has been shown to disrupt YAP oncogenic activity [110,111]. Other research regarding bevacizumab, a vascular endothelial growth factor (VEGF) inhibitor, has shown promise for *NF2*-associated schwannomas, meningiomas, and ependymomas [108,112]. Lastly, further proposed targets include the PD-1/PD-L1 axis, the chemokine receptor C-X-C chemokine receptor type 4 (CXCR4), and Ephrin receptor B2 [61,108,113,114,115]. As the function of Merlin and its interactions with various signaling pathways are better understood, additional molecular targets will be discovered, while clinical trials will continue to move forward to understand the utility of drugs for established targets.

## 6. Spinal Ependymoma with *MYCN* Amplification (SPE-MYCN)

Spinal ependymoma with *MYCN* amplification (SPE-MYCN) has recently been added as a new molecular category of ependymoma based on the updated 2021 CNS WHO classification, which was based upon several studies that have identified the presence of this molecular signature in a subgroup of highly aggressive SPEs. In 2001, Scheil et al. was the first to report on the amplification of *Myelocytomatosis-N* (*MYCN*), a gene located on chromosome 2p24.3 encoding a proto-oncogene transcription factor located in the cell nucleus that is critical for normal CNS development. This finding was based on a comparative genomic hybridization study of 26 ependymomas in 22 patients. Two cases of SPE-MYCN were identified with histological characterizations leading to a diagnosis of SP ependymoma WHO Grade III, one of which had relapse and intracranial metastases [116]. Later, Ghasemi et al., using DNA methylation analyses, observed *MYCN* amplification in a cohort of 13 tumors, of which 10 were WHO Grade III and three were WHO Grade II [117]. This series identified a strikingly worse prognosis than any other SP ependymoma subgroup, with a PFS of 17 months and a median OS of 7.3 years. These tumors were also unique in their location, favoring the cervical and thoracic spine, being predominately intradural and extramedullary. The presence of diffuse leptomeningeal spread, and dissemination was observed in 100% of cases. Further supporting the importance of this molecular marker was the retention of *MYCN* amplification in all recurrent tumors assessed, as well as the presence of MYCN protein overexpression by immunohistochemistry, suggesting malignant progression being driven by increased *MYCN* gene expression. Using a similar DNA methylation assay, two other studies recorded similar results, leading to a total of 27 published cases of ependymoma with *MYCN* amplification, which has led to the categorization of this subset of SPE [118,119]. Altogether, these studies demonstrate a slight predilection for the female sex with a mean age of diagnosis in the third decade of life. While these retrospective studies without prospective confirmation represent only a small number of patients, disallowing any clear incidence or strong epidemiological data to be extrapolated, what has thus been reported has called for this molecular marker to be further studied and used as a prognosticator of poor outcomes. *MYCN*, a member of the family of MYC oncogenes, plays a large role in neurogenesis and has been implicated in the genesis of various CNS malignancies such as neuroblastoma, pediatric glioblastoma, and medulloblastoma, as well as malignancies outside of the CNS such as leukemia and prostate cancer [120,121,122,123,124]. There are no current clinical trials targeting MYCN; however, there are several reported investigations assessing strategies for MYCN inhibition. Among these promising reports include inhibitors targeting histone deacetylase (HDAC), poly(ADP-ribose) polymerase (PARP), Auro A-kinase (AURKA), and Bromodomain and extraterminal domain (BET) proteins, as well as immunotherapy targeting through DNA vaccination [125].

## 7. Posterior Fossa Ependymoma (PFE)

Posterior fossa ependymoma (PFE) has been studied and classified by many studies. Initially these studies used gene expression and copy number profiles, and defined two groups of tumors: Groups 1 and 2 by Wani et al., and Groups A and B by Witt et al. and by Hoffman et al. [42,43,44]. The groups identified correlate across studies with Group 1/A associated with younger age and a more aggressive course compared to Group 2/B. With the introduction of DNA methylation profiling technology, additional studies confirmed these findings and made uniform the terminology for posterior fossa ependymoma Group A (PFA) and posterior fossa ependymoma Group B (PFB). These two groups are now part of the WHO 2021 classification [37,38,39,40,49,50,126]. PFE that are not PFA/B can be qualified as “other”, “not otherwise specified (NOS)”, which includes those that are not able to be molecularly analyzed, or “not elsewhere classified (NEC)”, which includes those with other molecular alterations. In an analysis of 35 PFE, 14 (40%) patients had PFA, 17 (48.6%) patients had PFB, and 4 (11.4%) patients were not classified into either, and thus should be presumed to be PFE, NOS/NEC [42]. Many studies characterize PFE based on their unique DNA methylation signatures [40,126,127]. As DNA methylation testing is not widely available, the use of an antibody against the trimethylated histone H3 at lysine 27 (H3K27me3) has been used to differentiate PFA from PFB [127,128,129,130]. There is a reduction or loss of H3K27me3 immunoexpression in PFA and persistent H3K27me3 immunoexpression in PFB [127,128,129,130].

Although the molecular characterization of PFEs has caused significant change in the categorization of these tumors, the DNA methylation results should be interpreted with care and in context with histology. There are many reasons why bioinformatics analyses (of any kind) should be interpreted with care, but this is beyond the scope of this article. Briefly, this starts with the quality of the tissue processed (time stored in formalin vs. fresh), the accuracy of tissue selected (tumor vs something else) and the algorithms used for data normalization. Similarly, the parameters, cut-off scores, and functions used for the analysis proper are of outmost importance. In the case of methylation there is no gold standard as of yet, and although the bioinformatic strategy used in the largest study published so far on brain tumors is now being commercialized, this algorithm was not sufficiently validated. Moreover, although this study included a large number of samples overall, when divided per rare entities the number of samples was small [131]. Therefore, methylation analysis, despite some opinions, is far from being “gold standard” for brain tumor diagnosis. For example, in an adult study, DNA methylation profiling of 38 IT tumors (seven WHO grade I SE, twenty-five WHO grade II ependymoma, and six WHO grade III anaplastic ependymoma) recategorized them as 24 PF-SE subgroup, 1 PFA subgroup, and 13 PFB subgroup; therefore this classification should be questioned as many ependymomas (*n* = 17 in this study) were classified as SE [37]. Similarly in another study, 11 IT ependymomas were classified by DNA methylation profiling as PF-SE [40].

PFEs are histologically grade 2 or 3, as described above in the SPE section (Figure 2D–F). As the molecular classification of PFE tumors is fairly novel, there are few studies describing specific subgroup location and radiographic characteristics. PFE is characterized as arising from the ependymal lining of the IVthV, often extruding out of the foramina of Luschka and Magendie [132,133]. PFE demonstrates heterogeneous enhancement, and approximately 50% demonstrate calcifications, with early evidence that calcifications primarily occur in PFA. Among patients with PFE, signs and symptoms related to CSF obstruction including headaches, nausea/vomiting, and lethargy are common, given their location in the IVthV [67,77].

## 8. Posterior Fossa Ependymoma Group A (PFA)

PFA is primarily a pediatric disease, with a median age of 3 years, and age ranging from 6 months to 58 years old across studies [37,40,41,42,127]. Adult cases make up a small percentage of the total cases. In studies with adult and pediatric PFA patients, 1–18.5% of patients are adults while the remainder are pediatric [40,41]. In a study of 134 adult PFE patients, 12% of the cases were H3K27me3-negative and presumed to be PFA [134]. PFA occurs more commonly in males, with a 65% to 35% male to female predominance. Aside from the aforementioned general PFE radiographic and clinical presentation, initial research on PFA demonstrates approximately two-thirds occur laterally and approximately one-third occurs medially [39,43].

In regard to ependymal tumors of the IT region, PFA has the poorest prognosis. Additionally, its prognosis is among the most dismal of all ependymoma subgroups. In comparing patients older than 10 years to those younger, the 5-year PFS and OS was 54% and 71%, which was not significantly different than patients younger than 10 years [39,40]. Although studies of purely adult PFA are uncommon, one such study had similar findings with H3K27me3-negative PFE patients having a 5-year PFS and OS of 44% and 80%, respectively [134]. This study showed that Ki-67 (MIB-1) index <10%, use of first-line radiotherapy, and GTR, were positive prognostic factors.

Overall, PFA tumors appear to have balanced genomes with the most frequent copy number variations being 1q gains (17.3–25%) and 6q losses (6.4–8.6%), both of which are negative prognostic indicators [40,135,136]. It is, rather, epigenetic changes such as the loss of H3K27me3 expression that lead to the PFA tumorigenesis [41,127,128]. There is increasing evidence that pathogenesis may be similar to that of diffuse midline gliomas (DMG) with *H3K27M* mutations and, in very rare instances (0.6–4.2% of cases), PFAs share this mutation [128,129,130,136,137,138]. In DMG, *H3K27M* mutations induce derepression of pro-oncogenic transcription factors through global reduction of histone 3 K27 trimethylation, H3K27me3. In addition to sharing this global DNA hypomethylation, PFA tumors have increased H3K27me3 enrichment at select genomic loci similar to that of *H3K27M*-mutant DMG [128].

**Figure 3:**
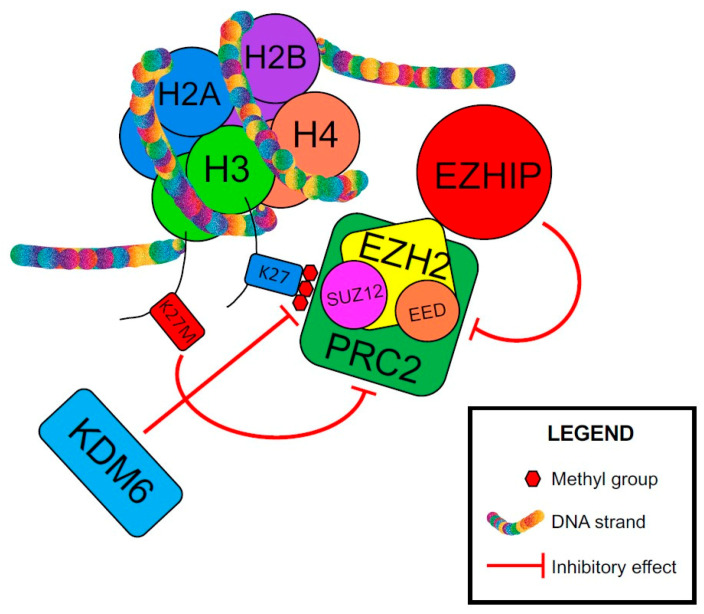
Mechanism of tumorigenesis in posterior fossa ependymoma Group A (PFA). EED—embryonic ectoderm development, EZH2—enhancer of zeste homolog 2, EZHIP—EZH inhibitory protein or Cxorf67, H2A—histone 2A, H2B—histone 2B, H3—histone 3, H4—histone 4, K27—lysine 27 on histone H3, K27M—methionine substitution for lysine on histone 3, KDM6—lysine-specific demethylase 6, PRC2—polycomb repressive complex 2, SUZ12—suppressor of zeste 12 homolog.

An important oncogenic driver in PFA is Cxorf67, or EZH Inhibitory Protein (EZHIP), and its interaction with polycomb repressive complex 2 (PRC2), a histone methyltransferase that primarily methylates H3K27. Both Histone H3K27M and Cxorf67/EZHIP have short sequences that bind and inhibit PRC2 [139]. These sequences bind to the Su(var)3-9/enhancer-of-zeste/trithorax (SET) domain of EZH2, which is part of PRC2 and inhibits its methyltransferase activity, mimicking the function of mutated K27M oncohistones and resulting in loss of methylation at residue K27 of Histone H3 (Figure 3) [136,139,140,141]. PFA ependymomas show increased expression of Cxorf67/EZHIP and absence of H3K27me3 [129,136]. Although rare, Cxorf67/EZHIP missense mutations (<10%) have been reported in PFA [136]. It was shown that these mutations do not alter the protein function [139]. Jain et al. hypothesized that these mutations increase EZHIP expression by altering the *cis*-acting gene regulatory elements [139]. Importantly, increased Cxorf67/EZHIP expression correlates with loss of H3K27me3 in PFE, and is mutually exclusive with *H3K27M* mutations [129]. The lack of trimethylation and the decrease in H3K27me3 level cause derepression/upregulation of PRC2 target genes, including genes involved in neurodevelopment, and likely contribute to PFA tumorigenesis [139,140,142]. Rarely, ATRX protein loss by immunohistochemistry (4–25%) has been reported in PFA [129,130]. Alpha-thalassemia, mental retardation, X-linked/death domain–associated protein (ATRX/DAXX) complex is involved in incorporating histone H3.3 at pericentric heterochromatin and telomers. ATRX loss leads to increased DNA damage and genomic instability [143].

There is evidence that the microenvironment plays a strong role in PFA growth and propagation [144,145,146]. An inflammatory state driven by chronic IL-6 and STAT3 expression differentiates this subtype from PFB [145,146]. The master regulator of IL-6, NF-κB, and its pathway are enriched in PFA tumors. Leucine zipper downregulated in cancer 1 (LDOC1) is a transcriptional repressor of NF-κB and is a key regulator of this pathway. LDOC1 gene expression is decreased in PFA in comparison to other pediatric brain tumors. Moreover, ependymoma cells treated with 5AZA-DC, a DNA methylase transferase inhibitor, upregulate LDOC1 expression and decrease IL-6 secretion. PFA growth is also dependent on hypoxia, and even transient exposure of PFA cells to ambient oxygen causes irreversible cellular death [144]. Hypoxia induces restricted availability of S-adenosyl methionine (SAM), a substrate for methylation including H3K27 methyltransferase EZH2, which leads to the PFA-characteristic globally diminished histone methylation and increased demethylation and acetylation of H3K27. Gene ontology analysis of PFA has demonstrated overexpression of genes associated with wound healing, angiogenesis (VEGF and HIF-1α signaling), and migration and adhesion (integrin signaling and extracellular matrix assembly), a pattern similar to the mesenchymal signature in glioblastoma [43,44]. Telomerase activity has been found to be a significant player in PFA pathogenicity [147]. Epigenetic hypermethylation of *hTERT* promoter and chromosome 1q gain were both strongly associated with telomerase reactivation in PFA.

Currently, there are no established targeted therapies for PFA. As PFA tumorigenesis appears similar to *H3K27M*-mutant DMG, medications directed at this cancer may also have therapeutic value in PFA [148]. Quantitative high-throughput screening of 2706 approved and investigational drugs, followed by testing on patient-derived xenograft models of *H3K27M-mutant* DMG, revealed the combination multihistone deacetylase inhibitor panobinostat and the proteasome inhibitor marizomib to have the highest therapeutic value and thus may have applicability in the PFA setting [149]. Given the limited genomic foci of H3K27me3 hypermethylation, DNA methylation inhibitors may be suitable targeted therapies. A study by Michealraj et al. demonstrated this to be the case with inhibition of histone lysine methylation, leading to diminished survival of resected PFA cell lines [144]. In a pilot study, 5-Azacytidine, a DNA methylation inhibitor, was used in pediatric recurrent PFE, and although it was found to be safe, it was not found to limit progression of disease at the study dosage [150]. Lastly, as previously discussed, EZHIP appears to be crucial in the oncogenesis of PFA [136,140]. Interrupting the interaction between EZHIP and EZH2 may block the oncogenic upregulation of PRC2 downstream genes, similar to targeting the residual activity of PRC2 or “detoxification” of Histone H3K27M as attempted for diffuse midline glioma [139]. A recent study showed that tumor cells with increased EZHIP expression suppress DNA repair and respond to PARP inhibitors, especially when associated with radiotherapy [151].

## 9. Posterior Fossa Ependymoma Group B (PFB)

Posterior fossa ependymoma Group B (PFB) tumors occur in adolescent and adult populations, with a median age of approximately 30 years and an age range of 2 to 65 years across studies [37,40,41,42,127]. These same studies demonstrated a slight predilection of disease towards the female sex. Studies with adult and pediatric PFB patients have demonstrated 75–81% of patients are adult aged, while the remainder are pediatric [40,41]. In the largest study of adult PFE patients, 88% of the cases were H3K27me3-positive and presumably PFB, while the other cases were H3K27me3-negative (PFA) [134]. In addition to the previously described clinical and radiographic findings, over 90% of the PFB subgroup tumors are located medially within the posterior fossa [43]. In comparison to PFA, patients with PFB have a better prognosis [39,40]. These tumors are rarely invasive or metastatic, and are unlikely to recur. Five-year PFS and OS was 83% and 98% in patients older than 10. In a study of adult ependymoma, H3K27me3-positive PFE patients were found to have a 5-year PFS and OS of 87% and 99%, respectively [134]. Similarly to PFA, prognosis of PFB is not affected by age at diagnosis but does benefit from GTR and Ki-67 <10% [38,39,134]. In contrast to PFA, both 5-year PFS and OS in PFB patients do not appear to be affected by first-line radiotherapy.

In comparison to the balanced chromosomal composition of PFA, PFB is characterized by a high degree of chromosomal instability, with many copy number aberrations present [40]; most notably, over 50% copy number losses in 6p and 6q, and over 50% copy number gains in 15q, 18p, and 18q [37,40]. However, thus far, no recurrent mutation has been found in PFB tumors [38,126]. Genomic investigation of PFB has demonstrated this subgroup to be more differentiated with an ependymal-like trajectory [126,152]. It is also characterized by hyperactivity of gene sets involved in sonic hedgehog (Shh) signaling, oxidative metabolism, and ciliogenesis/microtubule assembly (including *FOXJ1*) [40,43,44,152]. *FOXJ1* encodes Forkhead box protein J1, which is an important regulator of motile ciliogenesis, has been associated with Shh signaling, and is highly expressed in ependyma and choroid plexus [153,154]. The expression of FOXJ1 has been associated with several tumors including ependymal and gastric cancers, though this has primarily been decreased expression [154,155]. However, there are several instances of FOXJ1 overexpression in tumors, such as clear cell renal cell carcinoma and colorectal cancer, that merit further investigation [156,157]. In a colorectal cancer study, the overexpression of FOXJ1 significantly promoted nuclear translocation of β-catenin, an important factor in the Wnt-β-catenin pathway and in intestinal tumorigenesis. An adaptor protein NHERF1/EBP50 has been found to suppress the Wnt-β-catenin pathway-driven intestinal neoplasia [158,159]. Interestingly, NHERF1/EBP50 is a strong diagnostic marker for ependymoma in general, is an organizer of polarity structures such as ependymal cilia, and as further discussed in the ST-YAP1 section, has a domain PDZ-2 that binds to both β-catenin and YAP1 [51]. There are several medications that indirectly affect the Wnt-β-catenin pathway, but the development of directed small molecule inhibitors is still in its infancy [160,161,162]. More recently, a first in class NHERF1 PDZ1-domain inhibitor has been developed that addresses the PDZ1-domain role of membrane recruitment/displacement [163]. Although these medications are being developed for other indications, the combination of NHERF1 PDZ1 inhibitors with β-catenin inhibitors may have a role in future care of ependymal tumors.

## 10. Supratentorial Ependymoma (STE)

The new 2021 WHO Classification of CNS tumors included two main subsets of ependymomas located in the ST space: those that have *YAP1-fusions* (ST-YAP1) and those that have *ZFTA-fusions* (ST-ZFTA) [49,50]. The non-YAP1/ZFTA STE are best classified as “other” or “NOS/NEC”, and a histological grade can be offered. Further investigation will lead to other possible molecular subgroups. Additionally, as future studies begin to utilize these subcategories, further prognostic data will likely emerge. Earlier studies assessing prognosis of STE prior to molecular classification likely represent a heterogeneous population which include the now identified molecular subgroups, as well as tumors that would fall into the STE, NOS/NEC subgroup. Therefore, at the moment, no demographic or prognostic data are available for this subgroup, which remains, as of now, a subgroup of exclusion.

## 11. Supratentorial Ependymoma with *YAP1-Fusion* (ST-YAP1)

Supratentorial ependymoma with YAP1-fusion (ST-YAP1) is primarily a pediatric subgroup of ependymoma, with the median age of approximately 1 year and an age range from seven months to 51 years across studies [40,164,165]. Its occurrence in the adult population is extremely rare, though documented. Approximately 13–25% of ST-YAP1 cases occur in males [40,165]. Prognosis is significantly better than ST ependymoma with *ZFTA-fusion*, with most reports of 5-year OS at 100% [40,165]. The genomic origin of this tumor involves the fusion of chromosome 11 *YAP1* gene with chromosome X *MAMLD1* gene, though fusion with another gene, *FAM 118B* [40,165]. YAP1 or yes-associated protein 1, the oncoprotein of *YAP1* gene, is a downstream effector in the Hippo signaling pathway [69]. YAP interacts with various transcription factors in the nucleus, including TEAD (transcriptional enhancer factor domain) transcription factors, which increases expression of genes involved in cell proliferation [166]. YAP works with another co-activator, TAZ, (also known as protein Tafazzin encoded by gene *TAZ*) in a similar but non-redundant fashion to complex with TEAD to act on gene targets [167]. Preventing the interaction of YAP with TEAD transcription factors resulted in lack of tumor formation in mice [166]. In another mouse study, ectopic expression of YAP1 led to generation of tumors with molecular and ultrastructural characteristics of human ependymoma [168]. This suggests their interaction is critical in formation of ST-YAP1 tumors, and may be a therapeutic target. YAP1 effects do not lie solely in the Hippo pathway; but have been linked to several pathways including the Wnt-β-catenin pathway and a mechanotransduction pathway [169,170]. YAP also interacts with adaptor molecule NHERF1/EBP50, an in vivo tumor repressor for intestinal adenoma development and an organizer of polarity structures such as ependymal cilia [51,158]. In a primarily adult study by Georgescu et al., NHERF1/EBP50 immunoexpression was shown to be diagnostic in a majority of ependymal tumors but not of other CNS tumors. YAP activity has also been implicated in drug resistance in a number of cancers, including esophageal cancer, oral squamous cell carcinoma, urothelial cell carcinoma, and radiation resistance in glioblastoma and medulloblastoma [171]. YAP has been demonstrated to assist in the immune escape of tumor cells by enhancing programmed death ligand-1 (*PD-L1*) gene expression, thus attenuating T-cell activation [171,172].

In the setting of breast cancer cell cultures that depend on YAP/TAZ, the use of on-market drugs dasatinib, fluvastatin, and pazopanib inhibit nuclear location of YAP/TAZ [173]. Dasatinib is a tyrosine kinase inhibitor used to treat chronic myeloid leukemia, fluvastatin is a statin class drug that has been used in cell lines with antitumor effects, and pazopanib is a multi-kinase inhibitor of VEFG receptor-1, -2, and -3, PDGFRA, and c-kit used sarcoma and renal cell carcinoma treatment [174,175,176]. Oku et al. demonstrated that all three drugs induce phosphorylation of YAP/TAZ, and pazopanib also induces proteasomal degradation of YAP/TAZ [173]. In addition, a combination of these compounds was shown to reduce cell proliferation in YAP/TAZ-dependent breast cancer cells. As previously discussed in the SPE, Verteporfin has been shown to have anti-oncogenic effects through inhibiting YAP1 activity [110]. In a study using a mouse model with patient gastric cancer xenografts, verteporfin treatment was shown to inhibit cancer growth in vivo [177]. Based on these studies, targeting YAP and/or its co-activators will likely be the mainstay of therapeutic candidates in ST-YAP1 and likely in other cancers as well.

## 12. Supratentorial Ependymoma with *ZFTA-Fusion* (ST-ZFTA)

ST-ZFTA is the second main subgroup of ependymoma in the ST compartment and harbors the worst outcome [1]. ST-ZFTA has a predilection for the lateral and third ventricle, with most occurring adjacent to the ventricular system, but it is not uncommon for this subset of ependymoma to be extraventricular and cortically based [178]. While the median age of patients with this tumor subgroup is 8 years, the range is 0 to 69 years, with 23% being adults and a peak incidence in the sixth decade of life for those diagnosed in adulthood [34,40]. There is a sex predilection towards males. Microsurgical resection with radiotherapy is considered the mainstay of treatment [2]. With resection and radiotherapy, this subgroup harbors a dismal prognosis with 10-year PFS of approximately 20% and OS of approximately 50% [40]. The role of chemotherapy for this subgroup is yet to be established; however, the recent introduction of this ependymoma subgroup into the WHO classification of CNS tumors will likely bolster research into the underpinnings of the oncogenesis of this tumor [2].

On immunohistochemistry, L1 Cell Adhesion Molecule (L1CAM)/(CD171) corresponds to the presence of *ZFTA fusion* but is neither sensitive nor specific, while p65/double immunostaining has a 92% positive predictive value and a 100% negative predictive value [179]. ST-ZFTA was originally classified as ST-RELA based on the presence of the gene *REL-associated protein* (*RELA*) fusing with the gene Zinc Finger Translocation Associated (*ZFTA*), also known as *C11orf95*, being thought to be the distinguishing molecular characteristic. The reason for this change in nosology is based on several studies showing that both *RELA* and *ZFTA* fuse with various genes secondary to chromothripsis. However, ST-ZFTA demonstrates a consistent histomolecular entity following *ZFTA* fusion with or without *RELA*, thus underscoring the molecular importance of the presence of *ZFTA* fusion, as opposed to *RELA* [50,180,181,182,183,184]. It is reported that about 2/3 of ST ependymomas in children harbor this *ZFTA* fusion, and Pajtler et al. observed the presence of this genomic fusion in 72% of ST ependymomas [40,181]. While the landscape is vast for the possible fusion partners with *ZFTA*, the main fusion partners that have been identified and shown to be sufficient for tumorigenesis are *Mastermind Like Transcription Coactivator 2* (*MAML2*), *Nuclear Receptor Coactivator 2* (*NCOA2*), and *Nuclear Receptor Coactivator 1* (*NCOA1*) [182,183,184,185].

The introduction of the *ZFTA-fusion* subgroup was, in large part, a result of the landmark study by Parker et al. that identified the fusion of *RELA* with *ZFTA* in two thirds of ST ependymoma [181]. These tumors were found to involve a chromothripsis event with chromosome 11q13.1, the location of *ZFTA*. While the fusion of *ZFTA* has been established as the molecular marker distinguishing this subgroup, the function and role of *ZFTA* is still not completely understood. However, the introduction of this molecular subgroup has bolstered investigation into the function of *ZFTA*, and a recent study by Kupp et al. utilized a combination of transcriptomics, chromatin immunoprecipitation sequencing, and proteomics to elucidate the mechanism behind this gene [186]. This work has provided evidence that ZFTA tethers fusion proteins across the genome, modifying chromatin to an active state and enabling its partner transcriptional coactivators to promote uninhibited expression of various genomic targets. The work demonstrating the shuttling and enabling function of ZFTA serves as the foundation for future studies to develop targeted therapies attacking this protein. While the role of *ZFTA* is only recently being unraveled, *RELA*, the principal effector of canonical Nuclear factor-κB (NF-κB), has been well studied, and its tumorigenesis mapped out. RELA is normally located in the cytoplasm via nuclear factor of kappa light polypeptide gene enhancer alpha (IkBα)-mediated sequestration; however, upon external pressures, RELA protein is translocated to the nucleus to influence the NF-κB pathway [187,188]. Parker et al. demonstrated in a mouse model that the ZFTA-RELA fusion protein spontaneously translocates to the nucleus of neural stem cells to activate NF-κB target genes, which lead to the transformation of these cells into ependymoma [181]. Therefore, as ZFTA-RELA preferentially localizes to the nucleus, persistent activation of the NF-κB pathway is the proposed mechanism of oncogenesis. The NF-κB transcriptional regulator protein family is closely linked to cellular inflammation, and while constitutive activation of these proteins are often seen in human tumors, the rarity of mutations in members of this pathway has obfuscated research attempts to understand and identify targetable areas [189,190]. Additionally, Arabzade et al. showed through a autochthonous mouse tumor model that in addition to direct activation of canonical NF-κB, that ZFTA-RELA fusion protein binds to thousands of *PLAGL* (PLAG1 Like Zinc Finger 1)-enriched sites across the genome [191]. Moreover, ZFTA-RELA fusion protein recruits various transcriptional coactivators such as bromodomain-containing protein 4 (Brd4), histone acetyltransferase p300 (Ep300), and CREB-binding protein (Cbp), all of which are possible areas for future pharmacologic inhibition. Another promising target is *Fibroblast Growth Factor Receptor 3* (*FGFR3*). *FGFR3*, a gene located on chromosome 4 and responsible for bone development, has been shown to be overexpressed in an in-vitro model of ST-ZFTA. This study also demonstrated the efficacy of a broad range of FGFR inhibitors in inducing maturation in their invitro model, a therapeutic concept currently considered of high potential in pediatric cancers [192]. Lastly, immune checkpoint molecules are another possible target currently being investigated. Wang et al. uncovered that expression of PD-L1 independently predicts outcomes in patients with ST, extraventricular ependymomas [193]. T-cell exhaustion induced by overexpression of PD-L1 is a mechanism by which tumors evade immune-mediated clearance [194]. This observed overexpression in high-grade STEs that independently predicts outcomes further emphasizes the importance of this molecular trait and underscores the need for further studies assessing the efficacy of PD-L1 inhibitors in treating ST-ZFTA. While this pathway of oncogenesis is the subject of ongoing research, and has been further elucidated, the driver of chromothripsis causing this fusion is still not understood. It is theorized that in the pediatric setting, the deletion of *cyclin-dependent kinase inhibitor 2A/B* (*CDKN2A/B*) may play a role, as it influences the TP53 pathway which has been linked to chromothripsis in other tumors [40,195]. Given the established mechanism of oncogenesis, it is vital that inhibitors of RELA pathways, i.e., NF-κB signaling pathways, be further researched as viable therapies [188].

## 13. Conclusions

The novel description of the molecular origin of ependymoma has led to further understanding of this genetically diverse tumor. When compared to histopathological classification, the molecular categorization of ependymal tumors into 10 distinct subgroups has led to a more refined characterization of clinical course and potential molecular targets.

Given the unique profile of each ependymoma subgroup, future research must rely on molecular characterization rather than classical histopathological categorization alone. Genomic profiling of ependymal tumors is essential in the further elucidation of molecular targets. While many of ependymoma subgroups are aggressive, even the benign subgroups urgently need targeted therapies, as many tumors are not amenable to surgical resection without causing substantial disability. Research must continue to build on studies that have elucidated ependymoma oncogenesis, as well as investigate novel targets based on malignancies with similar gene ontology. An up-to-date clinical trial list regarding targeted treatments for ependymoma is available on “clinicaltrials.gov” (accessed on 9 November 2021). As the molecular underpinnings of ependymomas are further unraveled through the groundbreaking work of investigators in the field, it is expected that this list will continue to grow.

## Abbreviations

Note of Nomenclature: As the new WHO classification of tumors of the CNS is currently being published and could be released at any moment, it is noteworthy to mention that in this paper we keep WHO 2016 grade terminology (aka roman numerals) when we refer (or cite) to previously published studies that used 2016 classification criteria.

IV^th^V	Fourth ventricle
5AZA-DC	DNA methylase transferase inhibitor 5-aza-2′-deoxycytidine
AKT	Protein kinase B
ATRX/DAXX	Alpha-thalassemia, mental retardation, X-linked/death domain–associated protein complex
AURKA	Auro A-kinase
BET	Bromodomain and extraterminal domain
Brd4	Bromodomain-containing protein 4
*c-KIT*	Proto-oncogene encoding tyrosine-protein kinase KIT, CD117, or stem cell growth factor receptor
c-MYB	c-myeloblastosis
CAR T	Chimeric antigen receptor T cell
Cbp	CREB-binding protein
Cdc42	Cell division cycle 42
COX2	Cyclooxygenase-2
CNS	Central nervous system
CRL4	Cullin Ring Ubiquitin Ligase 4
CSF	Cerebrospinal fluid
CXCR4	C-X-C chemokine receptor type 4
EANO	The European Association of Neuro-Oncology
EED	Embryonic ectoderm development
EMA	Epithelial membrane antigen
Ep300	Histone acetyltransferase p300
EPHA7	Ephrin type-A receptor 7
ERK	Extracellular Signal-Regulated Kinase
ERM	Ezrin, radixin, moesin binding protein
EZHIP	EZH Inhibitory Protein or CXorf67
EZH2	Enhancer of zeste homolog 2
FAK	Focal Adhesion Kinase
FAM 118B	Family with sequence similarity 118 member B
FGFR3	Fibroblast growth factor receptor 3
FOXJ1	Forkhead box protein J1
GFAP	Glial fibrillary acidic protein
GTR	Gross total resection
H3K27M	Histone H3 lysine 27 to methionine mutation
H3K27me3	Trimethylated histone H3 at lysine 27
HDAC	Histone deacetylase
HE	Hematoxylin and eosin
HIF-1a	Hypoxia inducible factor 1 alpha
HOXA13	Homeobox A13
HOXB13	Homeobox B13
HOXC10	Homeobox C10
HOXD10	Homeobox D10
hTERT	Human telomerase reverse transcriptase
IkBα	Nuclear factor of kappa light polypeptide gene enhancer in B cells alpha
IL-6	Interleukin-6
IT	Infratentorial
KDM6	Lysine-specific demethylase 6
KIT	Tyrosine protein kinase or stem cell growth factor receptor or CD117
L1CAM	L1 Cell Adhesion Molecule
LATS1/2	Large Tumor Suppressor Kinase 1 and 2
LDOC	Leucine zipper downregulated in cancer 1
MAML2	Mastermind like transcription coactivator 2
MAMLD1	Mastermind like domain containing 1
MDM2	Mouse double minute 2 homolog or E3 ubiquitin-protein ligase Mdm2
MEF	Myocyte enhancer factor
MPE	Spinal myxopapillary ependymoma
mTORC1	Mammalian Target of Rapamycin Complex 1
MYCN	Myelocytomatosis-N
NCOA1	Nuclear receptor coactivator 1
NCOA2	Nuclear receptor coactivator 2
NEFL	Neurofilament light chain
NEC	Not elsewhere classified
NF-κB	Nuclear factor-κB
NF2	Neurofibromatosis type 2
NHERF1/EBP50	Na+/H+ exchanger regulatory factor/ezrin-radixin-moesin binding protein 50
NOS	Not otherwise specified
OS	Overall survival
PAK	P21 activated kinase
PARP	Poly (ADP-ribose) polymerase
PDGFRA	Platelet derived growth factor alpha
PFA	Posterior fossa ependymoma Group A
PFB	Posterior fossa ependymoma Group B
PFE	Posterior fossa ependymoma
PF-SE	Posterior fossa subependymoma
PFS	Progression-free survival
PI3K	Phosphoinositide 3-kinase
PIKE-L	Phosphoinositide 3-Kinase Enhancer-brain specific isoform
PLAGL	PLAG1 like zinc finger 1
PRC2	Polycomb repressive complex 2
Rac1	Ras-related C3 botulinum toxin substrate 1
RAC	Ras-related C3 botulinum toxin
RAF	Rapidly accelerated fibrosarcoma
Ras	Rat sarcoma virus
RELA	REL-associated protein
SAM	S-adenosyl methionine
SE	Subependymoma
SET	Su(var)3-9/enhancer-of-zeste/trithorax
Shh	Sonic hedgehog
SP	Spinal
SPE	Spinal ependymoma
SPE-MYCN	Spinal ependymoma with MYCN amplification
SP-SE	Spinal subependymoma
ST	Supratentorial
ST-RELA	Supratentorial ependymoma with RELA fusion
ST-SE	Supratentorial subependymoma
ST-YAP1	Supratentorial ependymoma with YAP1 fusion
STAT	Signal transducer and activator of transcription
STE	Supratentorial ependymoma
STR	Subtotal resection
SUZ12	Suppressor of zeste 12 homolog
TEAD	Transcriptional enhancer factor domain
*TAZ*	Gene encoding protein Tafazzin
VEGF	Vascular endothelial growth factor
WHO	World Health Organization
WP744	4′-O-benzylated doxorubicin analog
WP1066	JAK2/STAT3 inhibitor
WP1193	JAK2/STAT3 inhibitor
YAP1	Yes1 associated transcriptional regulator or YAP or YAP65
ZFTA	Zinc finger translocation-associated
